# A curated structural dataset of peptide–protein complexes reveals biases in existing datasets and principles of peptide binding

**DOI:** 10.1002/pro.70779

**Published:** 2026-09-15

**Authors:** Rahma Hamdani, Javier Delgado, Luis Serrano

**Affiliations:** ^1^ Centre for Genomic Regulation (CRG), The Barcelona Institute for Science and Technology Barcelona Spain; ^2^ Universitat Pompeu Fabra (UPF) Barcelona Spain; ^3^ ICREA Barcelona Spain

**Keywords:** binding energetics, covalent constraints, dataset curation, disulfide bonds, docking, hotspot residues, machine learning, peptide length dependence, peptide–protein interactions, protein–peptide complexes, secondary structure, structural curation, structural databases

## Abstract

Peptide–protein interactions are fundamental to many biological processes, and peptide design is gaining interest due to its therapeutic potential. This has led to the emergence of various structural databases, such as PepBDB and Peptipedia, that classify peptides as polypeptides with fewer than 50 amino acids. These databases provide valuable starting points for studying peptide recognition by protein partners and are widely used for machine learning applications, docking, and scoring functions. However, under such a length definition for peptides, we have very different cases that are likely to confound the analysis of peptide binding, including miniproteins, peptide–peptide complexes, intramolecular peptide disulfide bonds, intermolecular disulfide bridges, and proteins undergoing internal cleavage, such as serpins, as well as non‐natural amino acids and covalently bound cofactors. Here, we present a rigorous classification of peptide–protein complexes to generate datasets suitable for comparative energetic analysis with a focus on peptides that are unstructured in the absence of their target protein. The analysis of this dataset shows that peptide binding is typically driven by a small number of hotspot residues mainly enriched in aromatic and bulky hydrophobic side chains. Their number of hotspots and their spatial organization depend on peptide length, secondary structure, and covalent constraints. Short peptides rely on central anchor regions, whereas longer peptides distribute hotspots more broadly, with helices showing periodic spacing and β‐strands relying more on backbone‐mediated stabilization. Disulfide bonds further decrease the number of hotspots per peptide length by either pre‐organizing the peptide or acting as covalent anchors. This work provides a curated resource and general principles for peptide recognition. It highlights the importance of structurally classifying peptide–protein complexes to avoid bias in downstream computational and machine‐learning applications.

## 1 INTRODUCTION

Peptides exhibit structural uniqueness compared to small molecules and proteins, combining diversity and structural adaptability (Watt, 2006). Peptides mediate a wide range of biological processes, including signal transduction, transcription regulation, immune recognition, and interaction mediation (Chatterjee et al., 2013). In addition to their natural functions, peptides have attracted interest as therapeutics due to their small size, which enables them to target protein interfaces that are usually inaccessible and reduces production price.

Peptides interact with protein targets in a manner different from protein–protein interactions because of their unconstrained, flexible main chains, which allow them to adopt many conformations, unlike proteins, which come as pre‐folded, large units. These structural differences typically come with an entropic cost upon binding, which should restrict many conformations to a single bound pose (London et al., 2010). The peptide interface is typically shorter and more adaptable than a normal protein interface, relying on local backbone geometry rather than large, rigid surface complementarity. As a result, peptides are partially or fully disordered in the unbound state, with binding inducing a defined conformation.

This growing interest has motivated efforts in the peptide design field, ranging from experimental screening to rational in silico design. Central to these efforts is an understanding of how peptides bind their targets. This question is usually answered by using structural data to identify interaction motifs, characterize binding modes, and derive general principles for peptide design and structural optimization. Thus, the release of multiple structural databases of peptide–protein complexes, such as PepBDB (Wen et al., 2019), PepBank (Shtatland et al., 2007), PepBind (Das et al., 2013), and Peptipedia (Cabas‐Mora et al., 2024), that in general define a peptide based on its length and are widely used in peptide–protein interaction analysis or as training data for many predictive models.

However, defining a peptide solely based on sequence length is insufficient if the aim is to learn principles of peptide–protein binding and to engineer them rationally. Thus, there is no verification whether the short chain represents an actual independent peptide ligand not bound covalently, a cleaved fragment of a larger protein, a peptide assembly (such as helical coiled coil complexes), if it is covalently bound to the protein, or if it is a miniprotein with defined structure, or a crystallographic artifact. Moreover, some entries classified as peptide complexes lack a direct physical interface between the annotated peptide and the protein partner, because the interaction itself may be mediated by cofactors such as metals or sugars (lectins), which can further complicate interpretation. Finally, there is no filtering by sequence or structural homology. As a result, there is a significant degree of redundancy (e.g., MHC I complexes, Calmodulin peptide complexes), which could bias the analysis.

These inconsistencies introduce noise into peptide–protein structural datasets. When such datasets are used for statistical analysis or as training material for machine learning models, they can bias interaction patterns, distort energetic distributions, and inflate apparent diversity. Therefore, a rigorous structural validation beyond simple length thresholds is essential to construct a reliable peptide–protein interaction database.

In this work, we re‐evaluate a peptide–protein complex dataset derived from PepBDB (reported to have 13,489 complexes during the time of the download) using strict structural and interface‐based criteria. First, we removed misannotated fragments, incomplete complexes, cleaved proteins (e.g., serpins), low‐quality structures, and complexes crystallized at different times. We then classified the complexes into different groups. First, and the more important one, is a complex between a protein and a peptide that lacks a defined structure in the absence of its target protein. Then we formed groups from peptide assemblies, protein‐miniprotein complexes, disulfide‐containing peptides, or peptides bound via a disulfide bond to the protein. Thus, we generated a curated dataset comprising different peptide categories suitable for quantitative analysis and computational modeling. We have also analyzed the curated dataset to identify key factors that can guide peptide design tasks. Here, we present a systematic structural curation of peptide–protein complexes aimed at separating non‐covalent binding principles from cases involving covalent constraints or mis‐annotation.

## 2 RESULTS

### 2.1 A dataset of high‐resolution peptide‐protein complexes

To construct a comprehensive dataset of peptide–protein interactions, we initially collected structures from PepBDB (Wen et al., 2019), a widely used structural repository that contains experimentally determined peptide–protein complexes from the Protein Data Bank (PDB; Berman et al., 2000; Burley et al., 2025). PepBDB provides one of the largest curated resources for peptide–protein binding modes, containing 13,489 complexes, and has therefore become a common reference dataset for structural analyses and computational modeling of peptide interactions. In addition, peptide chain identities are explicitly annotated within PDB filenames, facilitating downstream analyses.

**FIGURE 1:**
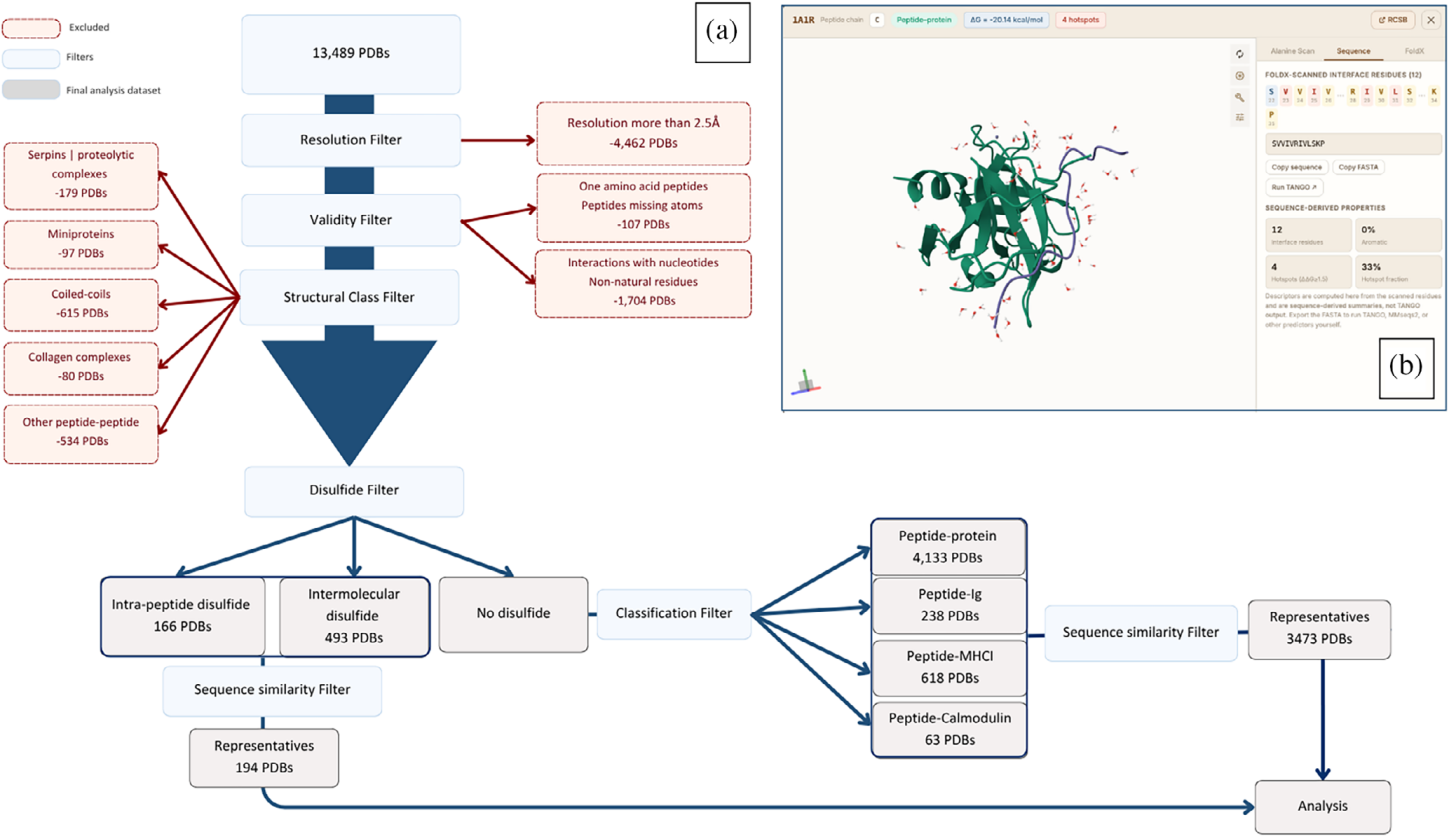
Multi‐Stage Filtering Strategy and Web‐Based Interface for Non‐Redundant peptide–protein Complexes. (a) Dataset Curation and Classification: Resolution, Validity, Structural Class, and Disulfide Filtering: A total of 13,489 entries were initially retrieved from PepBDB and filtered based on experimental resolution and sequence features, such as length and secondary structure elements, to form a set of unique peptide–protein complexes, which were then filtered for sequence similarity before being used in the rest of the analysis; (b) Interactive 3D Structure Visualization and Binding Interface Analysis.

Despite its utility, PepBDB includes entries that do not strictly correspond to canonical peptide–protein interactions. To obtain a dataset suitable for systematic energetic analysis, we applied a series of structural and biological filtering steps (Figure 1a). We first filtered the 13,489 entries based on experimental resolution, retaining only high‐quality structures with resolution ≤2.5 Å and removing 4462 lower‐resolution complexes. Peptides with incomplete chains or residues with missing or ambiguous atomic information were excluded. From the remaining set, we excluded assemblies that involved interactions with nucleic acids or contained non‐natural amino acids (1704 PDBs) to ensure compatibility with downstream energetic calculations.

The filtered dataset (7216 PDBs) was then classified based on structural features, including peptide and interacting protein lengths, secondary structure, and the presence of disulfide bonds. This resulted in six major categories: peptide–peptide complexes (further subdivided into coiled‐coils [615 PDBs], collagen‐like assemblies [80 PDBs], and other peptide oligomers [534 PDBs]), miniproteins that have a defined three‐dimensional structure (97 PDBs), peptides containing intramolecular disulfide bonds (166 PDBs), peptides forming intermolecular disulfide bonds with their protein partner (493 PDBs), and peptides lacking any disulfide constraints.

Within the subset of complexes without disulfide bonds, sequence‐based classification revealed strong enrichment in specific protein families, notably MHC class I (618 PDBs), immunoglobulins (238 PDBs), and calmodulin complexes (63 PDBs). To reduce bias arising from these overrepresented interaction types, these families were removed from the remaining dataset and subjected to sequence‐similarity clustering, retaining only one representative per cluster for subsequent analysis. Additionally, structures stabilized by metal ions, such as zinc, and that present a proper 3D fold were classified as miniproteins (97 PDBs).

As an additional quality‐control step, binding free energies (ΔG) were estimated using FoldX (Delgado et al., 2025). Complexes exhibiting extreme values (Δ*G* <−45 kcal/mol or Δ*G* >+45 kcal/mol) were found to correspond to cleaved protein fragments that remain associated with their parent structure (e.g., Serpins; Law et al., 2006). These entries were therefore reassigned to the category denominated serpins and proteolytic complexes (179 PDBs).

The remaining 4133 complexes were clustered by sequence similarity, retaining one representative per cluster to generate a non‐redundant dataset for subsequent energetic analyses. The same filtering was applied to MHC I, immunoglobulin, and calmodulin structures, yielding 3473 complexes, each containing a peptide expected to be unstructured in isolation. An equivalent redundancy filtering procedure was applied to complexes with disulfide constraints to ensure consistency across all subsets. Unless otherwise stated, this dataset is used for all subsequent analyses. A full classification of PepBDB entries is provided in Table S1.

### 2.2 Global energetic features of non‐disulfide peptide binding interfaces

#### 2.2.1 Binding interfaces are dominated by a limited set of aromatic and hydrophobic hotspots

The curated dataset facilitated the characterization of structural and energetic features that define the key features mediating peptide binding to protein interfaces. Our goal was to identify residue‐level determinants that govern peptide interactions and stabilization. By quantifying per‐residue energetic contributions with FoldX and mapping interface properties across complexes, we aimed to extract general principles relevant to peptide binding and design.

To this end, we first repaired all the PDBs using the *RepairPDB* command in FoldX. Then we performed a hotspot analysis using the FoldX alanine‐scanning command (see Section 5) for all peptide residues in contact with their binding protein. For each position, the energetic contribution to binding was estimated as the change in binding free energy (ΔΔ*G* kcal/mol) upon mutation to alanine predicted by FoldX (Delgado et al., 2025). Positive ΔΔ*G* values, therefore, indicate residues that contribute favorably to binding. The distribution of ΔΔ*G* values for all peptide residues (Figure S1) exhibited a mean of 1.52 kcal/mol and a standard deviation of 1.56 kcal/mol. Based on this distribution and given its proximity to one of the commonly used hotspot thresholds of 1.5 kcal/mol (Clackson & Wells, 1995), residues with ΔΔ*G* ≥1.5 kcal/mol were classified as hotspots for the remainder of the analysis.

**FIGURE 2:**
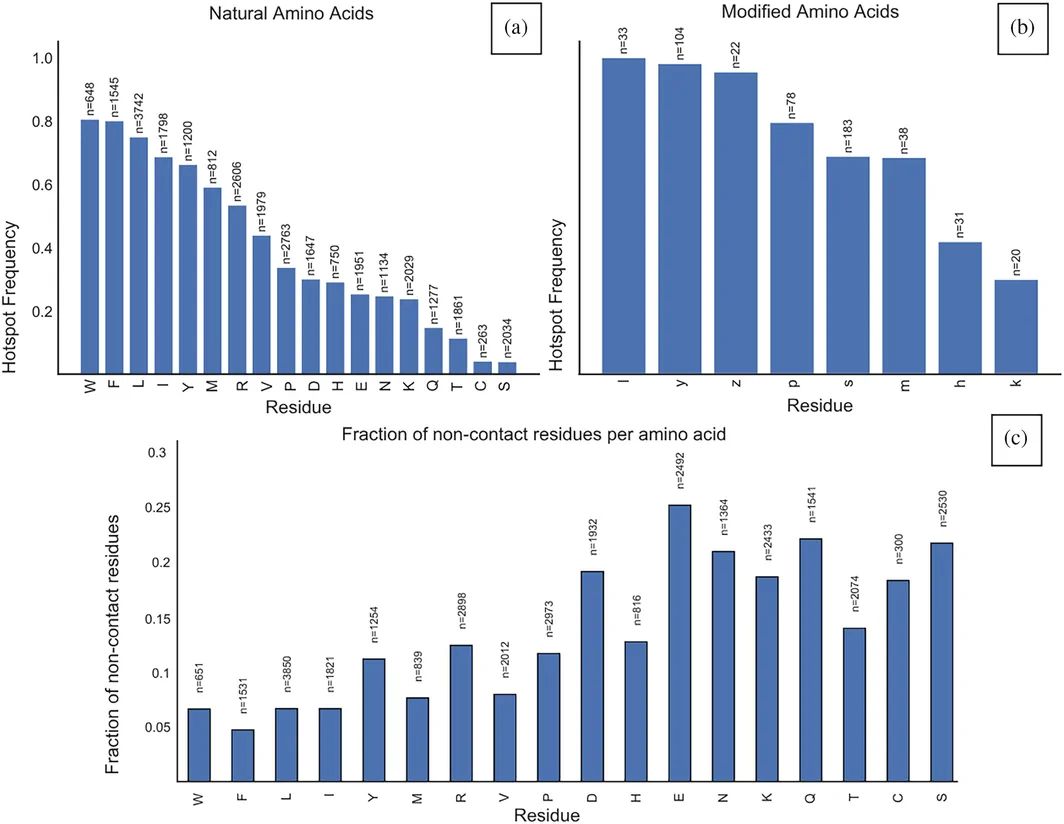
Amino acid‐specific hotspot propensity and non‐contact residue distribution in peptide–protein interfaces. (a) Fraction of residues in the contact interface and classified as hotspots for each natural amino acid, based on computational alanine scanning and a hotspot threshold of ΔΔ*G* ≥1.5 kcal/mol; (b) Hotspot frequency of modified amino acids found in peptide interfaces, including trimethylated lysine (l), phosphorylated tyrosine (y), sulfotyrosine (z), phosphorylated threonine (p), phosphorylated serine (s), dimethylated lysine (m), hydroxyproline (h), and monomethylated lysine (k); (c) Fraction of residues of each amino acid type that do not contact the target protein. Numbers above bars (n) indicate the total number of observations for each residue type.

We then examined whether specific amino acid types are preferentially enriched at hotspot positions as defined above. The analysis of hotspot frequency per amino acid type revealed differences in the tendency of residues to contribute critically to peptide binding (Figure 2a). Aromatic and bulky residues showed high hotspot enrichment, with tryptophan (W), phenylalanine (F), and tyrosine (Y) among the most frequent hotspot residues. Leucine (L), isoleucine (I), and methionine (M) were also highly represented among energetically significant positions, indicating a strong contribution of hydrophobic side chains to peptide–protein interactions (See Table S3 for frequencies). This enrichment is consistent with the central role of hydrophobic packing and π‐ π interactions in stabilizing peptide–protein interfaces, in which a limited number of deeply buried side chains often anchor the peptide within receptor pockets. An exception is arginine, which is quite often found as a hotspot.

In contrast, polar and small residues such as serine (S) and threonine (T) exhibited substantially lower hotspot frequencies, as they usually contribute more to structural or solvent‐exposed roles than to acting as primary binding drivers. Charged residues such as histidine (H), lysine (K), aspartate (D), and glutamate (E) displayed intermediate behavior, except for arginine, as mentioned above. Cysteine (C) showed one of the lowest hotspot fractions: consistent with its limited representation in non‐disulfide‐stabilized interfaces. We also looked at post‐translationally modified residues trimethylated lysine (l), phosphorylated tyrosine (y), sulfotyrosine (z), phosphorylated threonine (p), phosphorylated serine (s), dimethylated lysine (m), hydroxyproline (h), and monomethylated lysine (k), and (Figure 2b). Several post‐translationally modified residues are frequently found as hotspots, consistent with their ability to introduce strong electrostatic interactions (phosphorylation) or hydrophobicity (methylation) at the interface and to enhance binding at specific positions strongly.

When we look at the amino acids not contacting the target protein (Figure 2c), we see an almost mirror image of Figure 2a, with amino acids of lower binding energy being more frequently observed in non‐contact positions, highlighting the distinct compositional signatures of binding and non‐binding regions within peptide sequences.

Importantly, “non‐contact” refers here to residues that are not classified as part of the binding interface by FoldX according to the energy‐based criterion described in the Section 5. A purely distance‐based definition would not fully capture several energetically relevant effects, including long‐range electrostatic interactions involving charged residues, shielding of polar groups by bulky side chains, and solvent‐mediated interactions in which hydrogen bonding with water competes energetically with direct protein‐peptide contacts. Although our FoldX‐based calculations account for electrostatic interactions and solvation or desolvation effects, they do not explicitly capture water‐mediated hydrogen bonds bridging polar groups in the peptide and target protein. Future studies incorporating explicit water‐bridges networks would be valuable for understanding the full contribution of these residues to binding.

Overall, the distribution supports a binding model in which peptide interfaces are frequently stabilized by a small number of dominant hydrophobic or post‐translationally modified anchor residues embedded within a broader, more polar sequence context. While this analysis identifies which amino acid types most often contribute critically to binding, it does not address how these contributions are distributed along the peptide sequence. To overcome this limitation, we next studied the spatial organization of energetic contributions. We performed a position‐dependent energetic profiling across peptides of different lengths. This analysis allows us to determine whether hotspot residues are randomly distributed or preferentially localized to specific regions of the peptide, and whether this positional behavior varies with peptide length.

### 2.3 Peptide length drives a fundamental reorganization of the binding Interface

**FIGURE 3:**
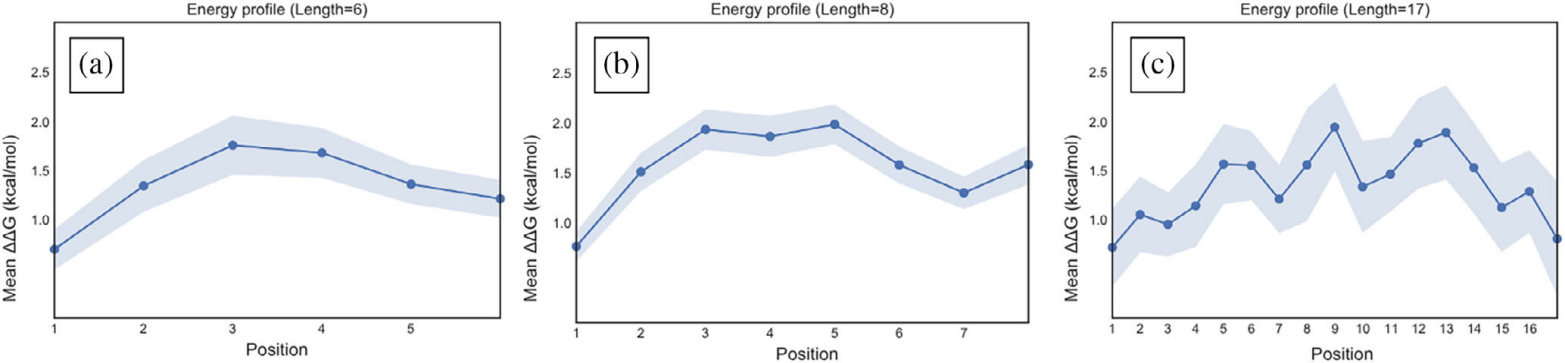
Position‐dependent energetic profiles across peptides of different lengths. (a) Mean ΔΔ*G* profile for peptides of length 6; (b) Mean ΔΔ*G* profile for peptides of length 8; (c) Mean ΔΔ*G* profile for peptides of length 17. For each peptide length group, the mean energetic contribution of residues at each sequence position was calculated from computational alanine scanning. Shaded regions represent the variability around the mean. Across all available peptide lengths, hotspot contributions are not uniformly distributed along the sequence (Figures S2 and S3).

Position‐dependent energy profiling (see Section 5) across all peptide lengths showed clear length‐based patterns in amino acid‐specific energy contributions, as determined by mutation to alanine. Short peptides (length 5 to 7) displayed predominantly uniform distributions, typically characterized by a higher contribution of the residues near the central positions (Figure 3a,b). In contrast, longer peptides showed progressively more heterogeneous profiles, with two main peaks emerging at lengths 8 and multiple distinct energetic peaks appearing in longer peptides, such as length 17 (Figure 3c).

These results indicate that short peptides tend to concentrate binding contributions within a confined medium region, whereas longer peptides distribute energy contributions across various spatially separated segments along the interface. In general, we observe in all peptides above 5 residues that the first hotspot residue starting from the N‐terminus is markedly enriched at a distance of 3–4 (median = 3), and the same thing happens starting from the C‐terminus (median = 3) (See Table S4). Additional hotspots did not follow a periodic spacing pattern but instead tended to cluster locally, with a median inter‐hotspot separation of approximately two residues (median = 2).

To determine whether hotspot distribution depends on peptide secondary structure, we examined inter‐hotspot separations across structural classes. While β‐sheet and coil peptides showed strong enrichment of short‐range separations (median hotspot separation ≤2 residues), helical peptides displayed a distinct spacing pattern (Figure S4). In helices, 40% of hotspot separations occurred at intervals of 3–4 residues, significantly higher than in β‐sheet or coil peptides (~19–21%; χ^2^ test, *p* = 1.19 × 10^−28^). These separations correspond to the 3.6‐residue periodicity of α‐helices (Emberly et al., 2002; Pauling et al., 1951), indicating preferential alignment of energetic residues along a helical section. Accordingly, helices exhibited reduced clustering compared to non‐helical peptides (Kruskal–Wallis test, *p* = 9.5 × 10^−28^; Figure S4). These observations support the idea that hotspot organization is shaped by secondary‐structure geometry, with non‐helical peptides favoring locally clustered energy patches. In contrast, helical peptides exhibit a periodic anchoring consistent with α‐helical topology.

### 2.4 Structural class predicts binding mode: How helices, coils, and strands stabilize different interfaces

To examine structural preferences in peptide–protein recognition, we classified both peptides and their associated protein partners according to their dominant secondary structure type (helix, β‐sheet, coil, or mixed), defined by the fraction of residues assigned to each class (see Section 5). A peptide or protein structure was considered to have a dominant secondary structure when more than 70% of its residues belonged to a given class; otherwise, it was classified as mixed. We then quantified the frequency of each pairing.

**FIGURE 4:**
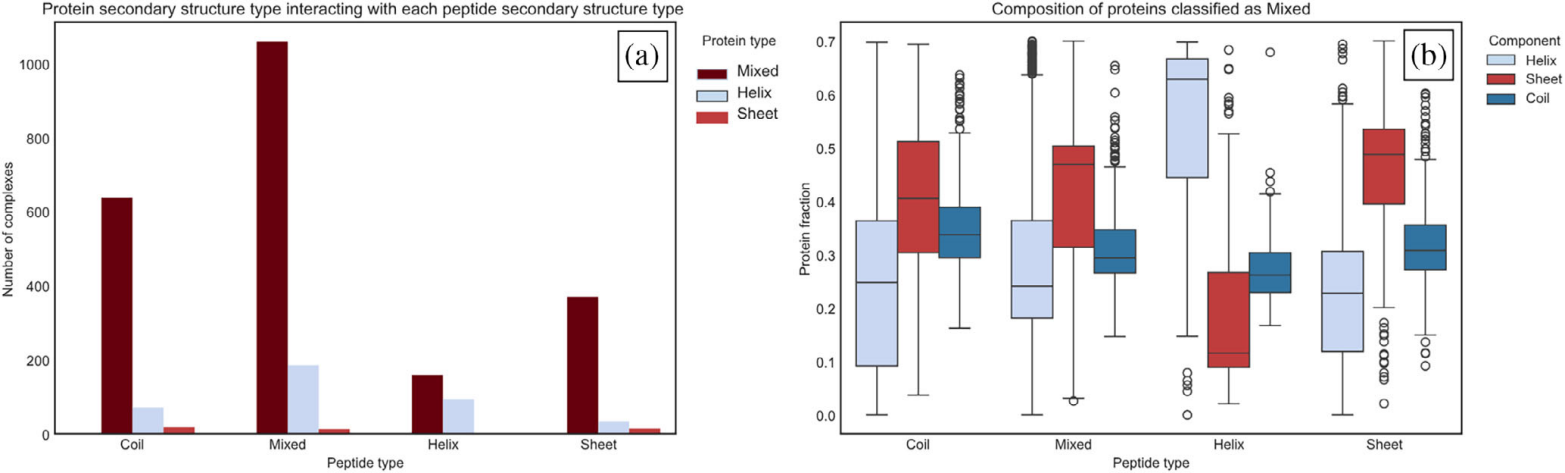
Protein secondary‐structure composition conditioned on peptide secondary‐structure class. (a) Number of complexes for each combination of peptide secondary‐structure class and protein secondary‐structure class; (b) Distribution of helix, β‐sheet, and coil fractions among proteins classified as mixed, shown separately for each peptide secondary‐structure class.

Figure 4a shows that, across all peptide classes, interactions occur predominantly with proteins assigned to the mixed category. This trend is strongest for mixed and coil peptides, which are found mainly in complexes with mixed proteins, with fewer interactions involving helix proteins and only rare cases involving β‐sheet proteins. Helical peptides also interact most often with mixed proteins, although a substantial fraction binds helical proteins as well. β‐sheet peptides follow the same general pattern, binding primarily to mixed proteins and less frequently to helix proteins, while associations with fully β‐sheet proteins remain uncommon. Overall, these data indicate that peptide‐binding interfaces are most often located in proteins with structurally heterogeneous local environments rather than in proteins dominated by a single secondary‐structure state.

Since the mixed protein class is the dominant partner for all peptide types, Figure 4b further explains its specific composition. Proteins in this category are not uniform: their relative helix, β‐sheet, and coil content vary depending on the peptide class they bind. Mixed proteins interacting with helical peptides are typically enriched in helical content, whereas those binding β‐sheet peptides tend to contain a larger β‐sheet fraction. In contrast, mixed proteins interacting with coil and mixed peptides show more similar combinations of helix, β‐sheet, and coil, consistent with a broader range of structural environments. Together, these results confirm that peptide recognition is strongly associated with proteins that present compositionally diverse interfaces (Petsalaki & Russell, 2008), while still maintaining compatibility between peptide conformation and the dominant structural features of the binding region.

As expected, there is a dependency of peptide length on the presence of helical conformations that start appearing in the 6–10 peptide length, increases in the 11–15 length and becomes similar in proportion to coils and β‐strands after that length (Figure S5), which is supported by the biophysical constraints on helix formation and stabilization (Zimm & Bragg, 1959).

### 2.5 Backbone vs. sidechain hydrogen bonds partition across structural classes

**FIGURE 5:**
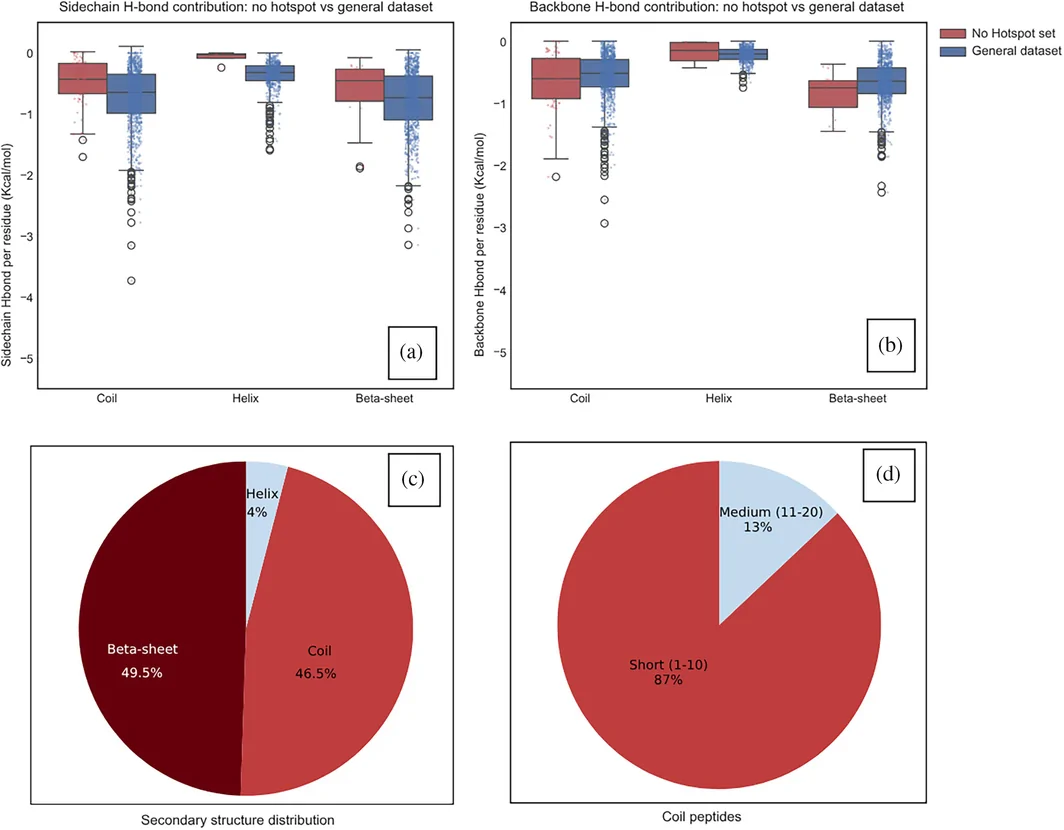
Hydrogen bonds energetic contributions across peptide secondary structure classes. (a) Distribution of sidechain hydrogen bond energy contributions per peptide across coil, helix, and β‐sheet conformations, comparing peptides without hotspots (no hotspot) and the general dataset; (b) Distribution of backbone hydrogen bond energy contributions across the same structural classes and datasets. Negative values indicate stabilizing interactions. (c) Secondary‐structure distribution of peptides in the no‐hotspot subset (ΔΔ*G* < 1.5 kcal/mol at all positions), showing strong enrichment in β‐sheet and coil conformations and depletion of helices; (d) Length distribution of coil peptides within the no‐hotspot subset, highlighting a strong bias toward short sequences (1–10 residues).

To assess how peptide secondary structure influences interaction energetics, we quantified backbone and sidechain hydrogen‐bond interaction energy contributions per residue for coil, helix, and β‐sheet peptides. As shown in Figure 5a, the side chain hydrogen bond contribution per residue is larger for coil and β‐sheet peptides (mean = −0.73 kcal/mol and mean = −0.79 kcal/mol, respectively) than in helical (−0.36 kcal/mol) peptides. The same happens for backbone hydrogen bond contributions (Figure 5b), where we see coil and β‐sheet peptides having larger contributions (−0.56 kcal/mol and −0.65 kcal/mol, respectively) than helical ones (−0.22 kcal/mol). If we compare side chain and main chain hydrogen bond contributions, we see that for β‐sheet peptides, the backbone hydrogen bond contribution is slightly larger than expected.

These results reflect key structural constraints: in α‐helices, backbone groups are largely engaged in intramolecular hydrogen bonds, limiting their participation in intermolecular interactions and shifting stabilization toward sidechain contacts. β‐strand peptides, by contrast, readily engage both backbone and sidechain hydrogen bonding, while flexible coil peptides rely predominantly on adaptable sidechain‐mediated interactions.

This is better seen in a small fraction of peptides that do not contain hotspot residues (148 complexes with ΔΔ*G* of mutation to alanine <1.5 kcal/mol at all positions). This no‐hotspot subset is composed almost entirely of β‐sheet and coil peptides, which accounted for 49.5% and 46.5% of cases, respectively. In contrast, helical peptides were rare, representing only 4% of the total (Figure 5c). Among coil peptides in this subset, the length distribution was also strongly shifted toward short sequences, with 87% ranging from 1 to 10 residues and only 10.4% ranging from 11 to 20 residues (Figure 5d). These results indicate that peptides lacking pronounced energetic hotspots are predominantly short and structurally enriched in β‐strand or coil conformations rather than helices. In coil peptides, however, we observe a clear inversion of interaction preference where backbone hydrogen bonds become more stabilizing than sidechain contributions (mean = −0.64 vs. −0.48 kcal/mol; *p* = 6.48 × 10^−3^). Also, β‐sheet peptides seem to have backbone hydrogen bonds significantly more stabilizing than sidechain contributions (mean = −0.87 vs. −0.64 kcal/mol; *p* = 8.22 × 10^−2^), indicating a dominant role for backbone‐mediated interactions in the absence of hotspots (Figure 5a,b).

Helical peptides do not show a significant difference between backbone and sidechain contributions (mean = −0.19 vs. −0.07 kcal/mol; *p* = 0.37), although interpretation is limited by their low representation (4%) in this subset. Together, these results reveal a structural dependence in the stabilization of binding in the absence of energetic hotspots. While coil peptides retain a preference for sidechain‐mediated interactions, β‐sheet and coil peptides exhibit a compensatory shift toward backbone‐driven stabilization, highlighting a distinct interaction mode that enables binding even in the absence of strongly contributing hotspot residues.

### 2.6 Covalent constraints in peptides reduce entropic loss upon binding

Covalent constraints, such as disulfide bridges, can influence peptide binding energetics, but their contributions differ depending on whether they are intramolecular or intermolecular. Intramolecular disulfide bonds, formed within the peptide, primarily affect binding through entropic stabilization of the peptide conformation. By restricting conformational flexibility in the unbound state, they reduce the entropic penalty upon binding and could pre‐organize the peptide into a binding‐competent conformation. In contrast, intermolecular disulfide bonds formed between the peptide and the protein directly contribute to binding by creating a covalent linkage across the interface, resulting in a strong, localized energetic contribution.

As a result, these two types of disulfide bonds are expected to impact hotspot distribution differently: intermolecular disulfides can act as dominant interaction anchors, potentially reducing the need for nearby additional hotspot residues but not after a certain distance, whereas intramolecular disulfides modulate binding more indirectly by stabilizing peptide structure without introducing a direct interfacial energy contribution and should have a reduced number of hotspots.

**FIGURE 6:**
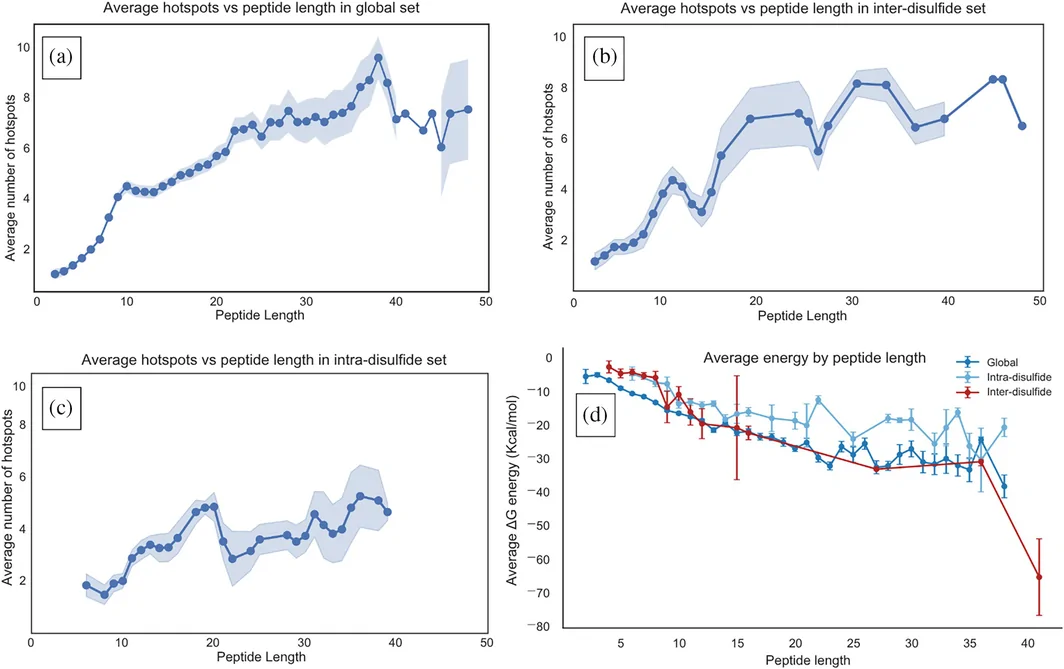
Correlating the hotspot number with peptide length across disulfide‐constrained and unconstrained peptides. (a) Average number of hotspots as a function of peptide length in the global peptide dataset; (b) Same analysis for peptides forming intermolecular disulfide bridges with their protein partners; (c) Same analysis for peptides containing intrapeptide disulfide bridges. Shaded regions represent the standard deviation around the mean hotspot count; (d) Plots representing the differences existing between three subsets in the values of average ΔG per peptide complex (disulfide constrained vs. non‐constrained).

These differences are highlighted by evaluating hotspot numbers with respect to peptide length across the different peptide classes (Figure 6a–c). In peptides lacking disulfide bonds (Figure 6a), the number of hotspots increases strongly with peptide length, showing a clear positive correlation (Spearman ρ = 0.62) and an approximately linear regime up to length around 40 amino acids (slope ≈0.14 and *R* ^2^: 0.77). After this length, the number of hotspots does not increase. This behavior indicates that, in unconstrained peptides, increasing chain length is accompanied by the accumulation of additional energetically important residues, consistent with the need to stabilize a larger and more flexible interaction surface.

Peptides forming intermolecular disulfide bridges (Figure 6b) display a similar but less regular trend (Spearman ρ = 0.72) with a slope of 0.15 and *R* ^2^ of 0.79. Although hotspot numbers generally increase with peptide length, the relationship is more variable. It exhibits local fluctuations, consistent with the presence of a strong covalent anchor that partially decouples hotspot accumulation from peptide length. Notably, these peptides tend to reach a lower plateau (~6–7 hotspots) at around 25 amino acids in length compared to unconstrained peptides (~6–7 hotspots) at around length 19–20, suggesting that the intermolecular disulfide bond reduces the requirement for additional stabilizing residues compared to peptides having the same lengths.

In contrast, peptides containing intramolecular disulfide bridges (Figure 6c) exhibit a weak relationship between peptide length and hotspot number (Spearman ρ = 0.43; Slope = 0.07 and R^2^: 0.56). Across this category, hotspot numbers increase to a length of around 18 amino acids and then remain relatively constrained (typically 4–5). This indicates that conformational pre‐organization imposed by intramolecular disulfide bonds reduces the need for multiple energetically dominant residues to stabilize binding.

To further investigate this effect, we examined the relationship between the number of intramolecular disulfide bridges and the binding free energy (ΔG) of each peptide–protein complex (Figure S6). We observed a trend toward more favorable (more negative) Δ*G* values with increasing numbers of disulfide bridges, indicating enhanced stabilization of the peptide fold. This supports the notion that structurally constrained peptides require fewer hotspots to achieve stable binding compared to less constrained peptides.

To more comprehensively assess the energetic consequences of these constraints, we compared average binding energies calculated with FoldX as a function of peptide length across the three peptide classes (Figure 6d). All peptide types show a general trend toward more favorable binding energies with increasing length. However, clear differences emerge between groups: peptides lacking disulfide bonds exhibit more favorable (more negative) binding energies across most lengths, whereas peptides with intramolecular disulfide bonds consistently display weaker predicted binding.

These observations are consistent with the concept of structural pre‐organization. Intramolecular disulfide bridges covalently constrain the peptide backbone, thereby reducing the conformational entropy of the unbound state and lowering the entropic penalty (–TΔS) upon binding. This pre‐organization allows the peptide to adopt a binding‐competent conformation with fewer energetically dominant residues (hotspots), as evidenced by the weaker correlation between peptide length and hotspot number (Spearman ρ = 0.43) and the plateau at only 4–5 hotspots. In contrast, unconstrained peptides must compensate for higher entropic losses by recruiting more hotspots (ρ = 0.62). Intermolecular disulfides, while also contributing to pre‐organization, act primarily as a strong covalent anchor at the interface, partially decoupling length–hotspot relationships and reaching a plateau earlier (~25 residues vs. ~19–20 for unconstrained peptides). However, a fraction of those containing intermolecular disulfide bridges display weaker binding energies for short peptides (up to length 12) (Figure 6b) because the disulfide bond will mainly be responsible for binding. Then they become like those that don't present disulfide bonds when the length increases. This apparent contradiction arises because, in short peptides, the disulfide bond dominates the interaction, reducing the relative contribution of non‐covalent interactions captured by FoldX.

### 2.7 Aggregation propensity of peptides subject to length and secondary structure elements

**FIGURE 7:**
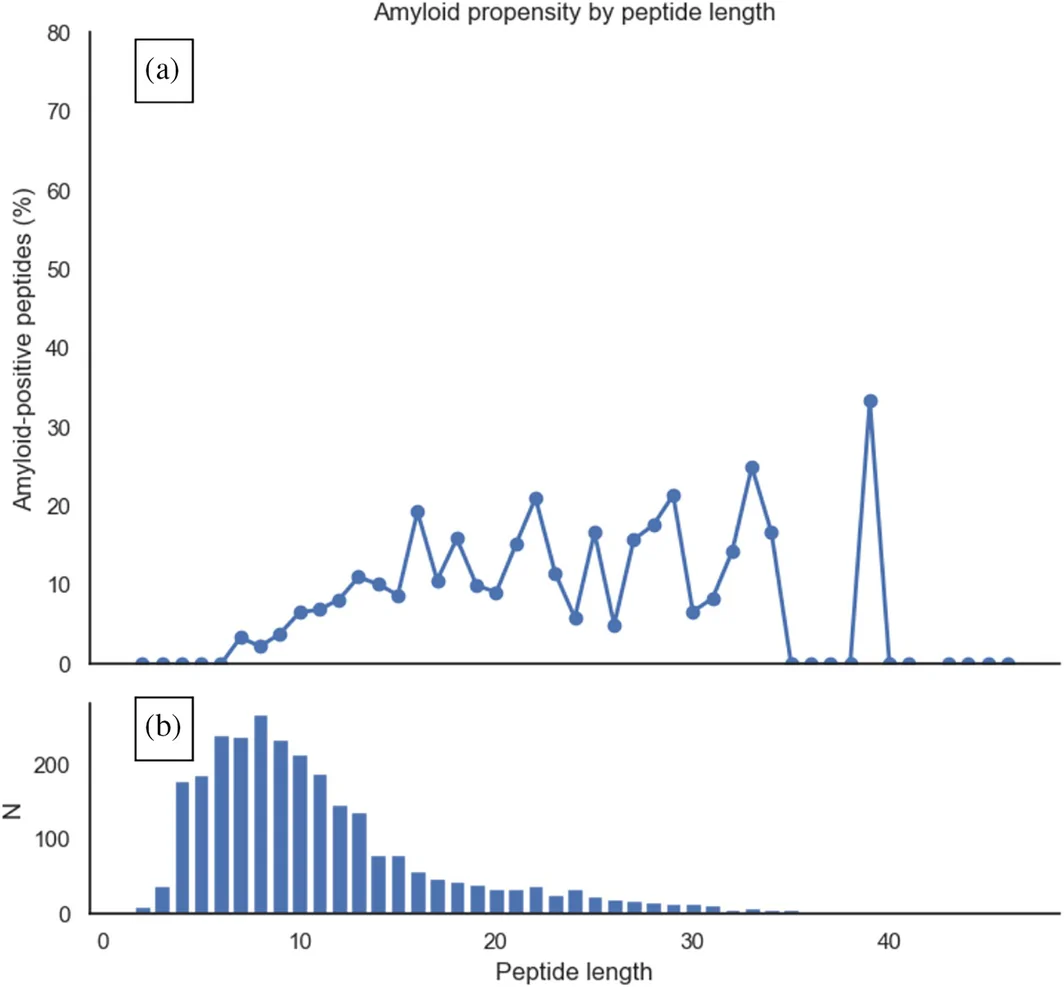
The evolution of the amyloid propensity with the length of the peptide. Plotting the percentage of the amyloid‐positive peptides by peptide length using the predictions of TANGO and the threshold of 5%. (a) A plot showing the amyloid‐positive rate per peptide length; (b) A bar plot showing the sample size per peptide length.

To further characterize intrinsic sequence properties associated with peptide binding, we examined how amyloid aggregation propensity varies with peptide length using TANGO predictions (Linding et al., 2004) (Figure 7). For each peptide, amyloid propensity was defined based on residue‐level aggregation scores, and a peptide was classified as amyloid‐positive if it contained at least five consecutive residues with TANGO aggregation values exceeding 5%.

Across the dataset, amyloid propensity shows a length‐dependent trend with notable variability (Figure 7a). Short peptides (length <6 residues) exhibit almost no amyloid propensity, with near‐zero fractions of amyloid‐positive sequences. As peptide length increases, the proportion of amyloid‐prone peptides rises gradually, reaching approximately 10–20% for peptides in the 10–25 residue range. This trend is consistent with the increasing likelihood of forming contiguous aggregation‐prone segments as sequence length increases.

For longer peptides (>25 residues), the fraction of amyloid‐positive sequences becomes more heterogeneous, with values ranging from ~20% to ~30% or higher. However, this variability coincided with a marked decrease in the number of peptides available in these length categories, as shown in the lower panel of Figure 7. Consequently, fluctuations observed at longer lengths are likely due to undersampling rather than true biological differences.

Overall, these results suggest that aggregation‐prone sequence features are rare in short peptides but increase as peptide length increases. This indicates that aggregation propensity is strongly selected against in small peptides, since they can readily aggregate. In contrast, longer peptides are more likely to contain local aggregation‐prone segments, but these stretches may coexist with residues that promote solubility or stabilize functional conformations, making aggregation propensity dependent on both sequence composition and structural context.

This length‐dependent accessibility of aggregation‐prone motifs is biologically relevant because aggregation can be nucleated by local flexible or unstructured regions that become solvent‐exposed. Such regions can support functional peptide–protein recognition by allowing the peptide to adapt to the surface of a binding partner, a common feature of intrinsically disordered or conformationally flexible protein regions. However, when exposed regions contain aggregation‐prone motifs, the same accessibility may also promote intermolecular contacts and self‐association (Ward et al., 2004). Thus, peptide binding and aggregation can be viewed as competing outcomes for exposed sequence segments: binding stabilizes the peptide in a functional complex, whereas unbound or weakly stabilized segments may instead form intermolecular beta‐sheet interactions (Wright & Dyson, 2015).

This competition is especially relevant for beta‐structure‐rich systems, including immunoglobulin‐like proteins and antibody light‐chain domains, where local destabilization or exposure of beta‐prone regions can promote oligomer formation and fibril growth (Blancas‐Mejia et al., 2018). Early oligomeric peptide assemblies may also act as recruiters for additional peptides or proteins, which result in amplifying aggregation through nucleation‐dependent growth mechanisms (Chiti & Dobson, 2017). In this context, preferred secondary structure provides an additional layer of interpretation: beta‐strand‐prone segments are generally more compatible with intermolecular beta‐sheet formation, whereas alpha‐helical tendencies may reduce aggregation risk in some contexts by favoring intramolecular or partner‐stabilized conformations. However, this relationship remains context‐dependent, since helical segments can also contribute to aggregation after partial unfolding, rearrangement, or exposure.

This analysis is also relevant for both peptide design and dataset usage. From a design perspective, it highlights the need to balance binding‐promoting features with aggregation risk, particularly for longer peptides. From a dataset perspective, it provides a way to identify and filter sequences with high intrinsic aggregation propensity that could bias computational modeling or machine learning analyses.

## 3 DISCUSSION

A central contribution of this work is the demonstration that structural peptide–protein repositories, although highly valuable to the community, are not automatically suitable for all energetic analyses or for the training of predictive models without prior curation. The entries grouped under the peptide–protein complexes umbrella can correspond to different biological and structural subgroups, including covalently bound peptides, cleaved protein fragments, coiled coils, and highly conserved and well‐represented protein family groups such as MHC I, which could bias the analysis. These different classes are informative on their own, but their inclusion in the general set could obscure the basic principles that govern non‐covalent peptide binding to target proteins. By applying a set of structural, sequence‐based, and energetic filters, we generated a dataset better suited for analyzing peptide–protein interaction mechanisms.

The complementarity of our work is further supported by comparison with Peptipedia (Cabas‐Mora et al., 2024), one of the largest sequence‐based peptide repositories. Exact sequence matching between our curated peptide sequences and the full Peptipedia entries showed that 1515 of 3757 unique peptide sequences overlapped (40.3% coverage); restricting both sets to only canonical sequences gave a similar result (1515 of 3293 unique sequences, 46.0% coverage). Peptipedia therefore captures a substantial fraction of the peptides represented here, but roughly half to 60% of our unique sequences are absent from it. This is expected given the different design of the two resources: Peptipedia is organized around sequence and biological activity, whereas the present dataset is organized around structures and per‐residue energetics which makes them complementary rather than redundant.

The analysis revealed that high‐impact residues or anchors dominate peptide binding to receptor proteins. Aromatic and bulky hydrophobic residues plus arginine are strongly enriched at these anchoring positions and are known as hotspots, while they are not enriched at all in non‐interacting positions. This composition supports a binding mode in which a small number of anchor residues provide a large fraction of the stabilizing energy. This observation has direct consequences for peptide design. Increasing the total number of contacts is unlikely to be sufficient on its own; instead, successful design should prioritize the placement of a few energetically dominant residues in positions that match the geometry and chemistry of the receptor surface, except for short peptides binding as part of a β‐sheet (see below).

Hotspot organization is dependent on peptide length and its secondary structure composition. In general, hotspot residues are not found in the first or last 3–4 residues of a peptide. As a result, in short peptides, they are mainly localized in the center. In contrast, longer peptides have hotspots distributed along the peptide length, except for the first and last residues, as indicated above. In parallel, spacing between hotspot residues is not arbitrary. Initially, helical peptides appeared to display the expected 3–4 residue periodicity characteristic of α‐helical geometry. However, direct comparison of energy profiles for helical versus non‐helical peptides of the same length (17 residues; see Figure S7) reveals that this ~3–4 residue periodicity persists equally in both groups. This observation indicates that periodicity is not driven by helical structure per se but rather reflects a more fundamental physical principle: the characteristic end‐to‐end distance predicted by random‐walk polymer physics (R_ee ~c ≈4.1 residues for length‐17 peptides; Alston et al., 2023). In other words, while helical peptides naturally display this spacing, it is not only because they are helical, but also because all peptides, helical or not, follow polymer chain statistics that govern how they can distribute binding contacts along their length. Notably, coil and β‐strand peptides show tighter 1–2 hotspot clustering, reflecting their different polymer conformations (see Data S1 for detailed random‐walk analysis).

Furthermore, the peptide's secondary structure strongly influences the type of stabilization these highly flexible molecules exhibit. In fact, coil and mainly helical peptides rely strongly on sidechain‐mediated hydrogen‐bond contributions when β‐strand peptides tend to favor more backbone interactions. This distinction becomes particularly informative in the subset of peptides lacking strong hotspots, where β‐strand peptides appear capable of compensating for the lack of anchors through backbone‐driven stabilization. Disulfide bonds introduce an additional stabilizing factor. Intrapeptide disulfides are consistent with conformational pre‐organization, reducing the need to accumulate multiple dominant hotspots. In contrast, intermolecular disulfides act as direct covalent anchors across the interface and partially substitute for additional non‐covalent stabilizing contacts.

The conclusions reached should be interpreted in the context of several limitations. First, the energetic values analyzed here are FoldX‐derived estimates based on bound structures and alanine substitutions and therefore reflect relative energetic contributions rather than experimental absolute binding affinities. Second, despite redundancy reduction, the dataset still reflects the availability and composition of solved structures in the PDB, including the reduced sampling at longer peptide lengths. Third, the present analyses are based on bound‐state models and therefore do not explicitly capture all aspects of coupled folding and binding, solvent dynamics, or kinetic effects. Even with these limitations, the curated resource provides a more appropriate benchmark for studying peptide recognition. It highlights the risks of deriving general rules or machine‐learning models from unfiltered structural repositories.

Finally, through data processing, we provide a summary table that allows users of PepBDB to extract and use peptide classes tailored to specific applications. For example, users interested in linear peptides, disulfide‐constrained peptides, or specific structural classes, such as MHC I, can directly access the relevant subset without additional curation. Also, this analysis makes downstream computational applications easier, particularly the extraction of features (e.g., the aggregation rate or the hotspot separation), which is suitable for training machine learning models of peptide binding affinity or hotspot prediction.

## 4 CONCLUSION

This analysis established a curated dataset of 4133 high‐resolution peptide–protein complexes represented in 2789 non‐redundant clusters of unconstrained peptides, designed to enable systematic analysis of peptide binding energetics and structural organization. The dataset shows that peptide recognition is typically driven by a limited set of hotspot residues, enriched in aromatic and bulky hydrophobic side chains, whose number and spacing depend on peptide length, secondary structure, and covalent constraint. Short peptides usually rely on compact anchor regions; longer peptides can engage targets through multiple energetically important segments; helical peptides exhibit periodic hotspot spacing; and disulfide bonds reduce the need for extensive hotspot accumulation by pre‐organizing or covalently anchoring the peptide. Beyond providing a refined resource for computational studies, this work shows that careful structural curation is essential for extracting robust design principles and for avoiding bias in downstream machine‐learning applications.

## 5 MATERIALS AND METHODS

### 5.1 Alanine mutagenesis using FoldX

Computational Alanine scanning was performed on all peptide residues using FoldX version 5.1 (Delgado et al., 2025). For the different complexes, the peptide chain is identified using the parsing provided by the authors of the original PepBDB dataset. Then, each position along the peptide chain was mutated to alanine individually using the *BuildModel* command. Mutations were introduced one at a time, keeping the receptor structure unchanged and the positions around the mutations unmoved by setting *moveNeighbours = False*. For each mutant structure, the binding interaction energy between the peptide and its binder was calculated using the *AnalyseComplex* command in FoldX.

### 5.2 Contact definition

FoldX identifies relevant contacts between peptide and protein through an energetic filter. For each residue, the energy of its side chain is computed first within the complex context, and then again after isolating the chain to which that residue belongs. If the two energy values differ, the residue receives an energetic contribution from the partner chain and is classified as an interface residue in contact with it; if they are identical, the residue is classified as non‐contacting. This approach captures all interaction types and ensures that residues are counted as interface contacts only if they make an actual energetic contribution across the interface.

### 5.3 Prediction of the change in binding free energy (ΔΔG)

The change in binding free energy (ΔΔG) was computed as follows: ΔΔG = ΔG_binding(Ala‐mutant) − ΔG_binding(wild‐type). Positive ΔΔG values indicate destabilizing mutations and identify residues that favorably contribute to binding. Hotspots were defined as residues whose mutation to alanine results in ΔΔG ≥1.5 kcal/mol. This alanine‐scanning procedure was applied systematically to all peptide positions across the curated dataset to generate position‐based energetic profiles. All calculations are fully reproducible using FoldX versions 5.1 (https://foldxsuite.crg.eu/) with the *AnalyseComplex* command parameters specified here.

### 5.4 Cleaning the peptide–protein complexes dataset

To construct a structurally consistent dataset, we implemented a multi‐step curation and refinement workflow (see Figure 1a). Starting from 13,489 entries in PepBDB, successive quality filtering, structural classification, and redundancy reduction resulted in a final set of 3473 non‐redundant peptides–protein complexes (both disulfide‐constrained and non‐constrained).

#### 5.4.1 Filtering out bad resolution and non‐valid peptide structures

As an initial refinement step, structures were filtered based on experimental resolution. Complexes with a resolution worse than 2.5 Å were excluded to ensure sufficient structural precision for downstream energetic calculations using FoldX. In addition, single‐residue peptides and invalid peptide chains were removed, as they do not provide meaningful information about peptide–protein binding interfaces.

Additional structural integrity checks were performed to exclude entries with incomplete or ambiguous atomic information. Complexes containing residues annotated as “UNK” were removed, as these lack defined chemical identities and are incompatible with reliable energetic evaluation. Structures with incomplete peptide chains, including broken sequences or missing segments, were also excluded. Furthermore, entries in which residues were represented only by Cα atoms, or where one of the interacting chains was missing, were discarded, as these lack the atomic detail required for accurate modeling of peptide–protein interactions.

#### 5.4.2 Removing the peptides containing non‐natural amino acids and the peptides binding nucleic acids

To enable consistent computational mutagenesis using FoldX's pipeline, complexes containing non‐naturally modified amino acids not supported by the FoldX force field were excluded. This step ensured uniform energetic evaluation across all the remaining structures. Furthermore, complexes in which the peptide was interacting with nucleic acids rather than with a protein binder were removed. Since the focus of our study is peptide–protein specific interactions, the inclusion of peptide–DNA or peptide–RNA assemblies would introduce fundamentally different interaction principles that could bias the dataset.

#### 5.4.3 Separating cleaved protein fragments and serpin‐derived peptides from the dataset

During the dataset inspection, a subset of complexes displayed extremely favorable interaction energies, with predicted ΔG values more negative than −45 kcal/mol. Manual examination of these outliers showed that many did not correspond to what we define as peptide–protein complexes, but rather to cleaved protein fragments or peptides derived from serpin‐like complexes and other internally cleaved proteins. In such cases, the shorter chain (the one considered the peptide) represented a large proteolytic fragment that retained extensive contacts with the original protein fold, leading to artificially strong interaction energies that are not representative of typical peptide–protein interactions.

#### 5.4.4 Structural classification and family bias control

The remaining complexes were further categorized based on sequence and structural features. A first level of classification identified peptide–peptide assemblies, in which both interacting chains are of comparable length and form symmetric or repetitive structures. These complexes were distinguished from classical peptide–protein interactions and further subdivided into structural classes, including coiled‐coils, collagen‐like assemblies, and other peptide–peptide architectures. Because these systems are governed by distinct structural principles, including periodic sequence patterns and extensive packing interactions, they were treated as a separate category throughout the analysis.

In addition, a subset of small, independently folded peptides stabilized by internal interactions or metal coordination was identified and classified as miniproteins. These structures, often stabilized by disulfide bonds or metal ions such as zinc, exhibit a compact fold that differs from that of typical linear binding peptides and were therefore classified separately.

Sequence‐based classification of receptor proteins within the remaining peptide–protein complexes revealed a strong enrichment of specific families, notably MHC class I complexes, immunoglobulins, and calmodulin‐binding systems. Because these families exhibit highly conserved binding modes, they were separated from the rest of the dataset to prevent overrepresentation from biasing global trends. Each group was treated independently and subjected to redundancy reduction before downstream analyses.

#### 5.4.5 Disulfide bridge

To further characterize structural constraints within the dataset, disulfide bridges involving peptide residues were determined using the *Disulfide* command implemented in FoldXpro. This command predicts both existing and potential disulfide bonds based on geometric criteria, including α_Carbon‐α_Carbon and β_Carbon‐β_Carbon, dihedral angles, and sulfur–sulfur bond length constraints, combined with an energetic evaluation of the modeled disulfide configuration. The procedure allows the identification of intramolecular (within the peptide) as well as intermolecular (peptide–protein) disulfide bridges and the peptides that belong to both classes.

This analysis revealed distinct structural categories. A subset of peptides was stabilized by intrapeptide disulfide bonds, thereby constraining their conformational flexibility before binding. Another subset formed intermolecular disulfide bridges directly linking the peptide to its protein, introducing covalent stabilization across the interface. Finally, complexes lacking any peptide‐associated disulfide bond form a fourth category, representing unconstrained peptides.

#### 5.4.6 Redundancy reduction and sequence clustering

To minimize overrepresentation of similar complexes, redundancy was reduced using sequence‐based clustering with MMseqs2, with sequence similarity filters of 90% for proteins and 80% for peptides (Steinegger & Söding, 2017). Clustering was not limited to protein sequences alone but included both chains of the complex. Protein chains and peptide sequences were clustered independently, yielding separate representative clusters for proteins and peptides. The resulting cluster assignments were then combined to define full complex clusters, such that each complex was uniquely identified by the combination of its receptor cluster and peptide cluster.

This strategy ensured that redundancy was reduced at the level of the entire peptide–protein complex rather than only at the receptor level. As a result, complexes with highly similar receptors but distinct peptides were retained as independent representatives, while assemblies with both similar receptors and peptides were grouped. Cluster sizes were quantified, and one representative per complex cluster was selected for downstream energetic analysis.

This full‐complex clustering approach substantially reduced dataset redundancy while preserving structural and sequence diversity across both binding partners. The resulting clusters' representatives were used in the downstream energetic analysis.

Note that the redundancy reduction for the sake of the comparison with Peptipedia used clustering on the peptide sequence alone since Peptipedia contains no information on binding partners.

### 5.5 Secondary structure prediction using FoldX

Secondary structure information for the residues composing the peptides was obtained using the *SequenceDetail* command in FoldX. This command analyzes the structural coordinates of each complex and outputs residue‐level information, including the secondary structure element assigned to every amino acid in the sequence. The secondary structure symbols follow the standard DSSP notation (Hekkelman et al., 2025): H for α‐helix, E for β‐strand, G for 31_10_‐helix, I for π‐helix, T for hydrogen‐bonded turn, S for bend, and C for coil or unstructured regions. These residue‐level annotations were subsequently grouped to determine the dominant secondary structure content of each peptide, which was then used for structural classification and comparative analysis across peptide–protein complexes. The secondary structure class of the peptide or protein corresponds to the secondary structure element that dominates 70% of the amino acids in the peptide. If no secondary structure class is dominant, the peptide's class is marked as mixed.

### 5.6 Hydrogen bond energy prediction

Energetic contributions at peptide–protein interfaces were extracted from the output generated by the *AnalyseComplex* command from FoldX. This analysis computes the interaction energy between binding partners into individual energetic terms, including hydrogen‐bond contributions from both the sidechain and backbone. From the resulting output files, backbone and sidechain hydrogen bond energy terms were extracted for each complex. Backbone hydrogen bonds correspond to interactions involving peptide backbone donor or acceptor atoms, whereas sidechain hydrogen bonds involve interactions mediated by amino acid sidechains. Energies were subsequently normalized per residue to enable comparison across peptides of different lengths and secondary structural classes.

### 5.7 Amyloid propensity prediction using TANGO

Amyloid aggregation propensity was evaluated for all peptides using the TANGO algorithm (Fernandez‐Escamilla et al., 2004), which predicts residue‐level aggregation tendencies based on physicochemical principles. For each peptide–protein complex, the peptide sequence was extracted from the PDB structure using the chain identifier encoded in the file name. Non‐canonical residues were excluded from the analysis, as TANGO is parameterized for the 20 standard amino acids and does not provide reliable predictions for modified or uncommon residues. Including such residues could therefore introduce undefined or biased aggregation scores.

Sequences were analyzed in their linear form, without incorporating structural constraints, as TANGO is designed to estimate intrinsic aggregation propensity solely from sequence composition under unfolded or partially unfolded conditions. This approach allows comparison across peptides independently of their bound conformations and isolates sequence‐driven aggregation tendencies from structural stabilization effects imposed by the protein environment.

TANGO calculations were performed under standard reference conditions (pH = 7.4, temperature = 298 K, ionic strength = 0.1 M), with both N and C‐termini treated as unprotected. Residue‐level aggregation scores were obtained for each sequence, corresponding to the predicted probability of aggregation at each position.

To assess length‐dependent trends, peptides were grouped by sequence length, and the fraction of amyloid‐positive peptides was calculated for each length category. The number of peptides contributing to each category was also recorded to account for uneven sampling across lengths.

## AUTHOR CONTRIBUTIONS

**Javier Delgado:** Conceptualization; methodology; supervision; writing – review and editing; data curation. **Rahma Hamdani:** Investigation; writing – review and editing; methodology; validation; visualization; writing – original draft; data curation; formal analysis; project administration. **Luis Serrano:** Conceptualization; methodology; writing – review and editing; supervision; data curation; project administration.

## FUNDING INFORMATION

This study was supported by funding from “Ayudas para contratos predoctorales para la formación de doctores/as 2022” (FPI: PRE 2022‐101389), the Fund for Scientific Research Flanders (FWO, SBO grant S000722N), ERC LUNG‐BIOREPAIR (101020135) ERC Advanced Grant (AdG). We also acknowledge the support of the Spanish Ministry of Science and Innovation through the Plan Nacional (PID2021‐122341NB‐I00), the EMBL partnership, the Centro de Excelencia Severo Ochoa (CEX2020‐001049‐S, MCIN/AEI/10.13039/501100011033), and the Centres de Recerca de Catalunya (CERCA) Programme/Generalitat de Catalunya.

## Supporting information


**TABLE S1.** Table containing the classification of all entries of the PepBDB.
**TABLE S2.** Table illustrating the change in binding free energy (ΔΔ*G*) upon mutations to alanine along the chain of the peptide.
**TABLE S3.** Frequency of Hotspots per amino acid type. This table reports the number of hotspot occurrences for each wild‐type residue observed in the dataset, together with the total number of mutations and the resulting hotspot fraction.
**TABLE S4.** First hotspot position with respect to N‐termini and C‐termini.
**FIGURE S1.** Histogram of ΔΔ*G* values obtained from alanine scanning across the curated peptide dataset. The vertical dashed line indicates ΔΔ*G* = 1.5 kcal/mol.
**FIGURE S2.** Positional sampling across peptides. Number of alanine mutations available per position for representative peptide lengths (6, 8, and 17 residues). This illustrates the positional coverage of the dataset used for the energy profiling analysis. The numbers change across positions because we don't consider glycine‐to‐alanine and alanine‐to‐alanine mutations.
**FIGURE S3.** Positional ΔΔ*G* profiles by peptide length. Mean ΔΔ*G* values across sequence positions for peptides grouped by length. Each panel corresponds to a specific peptide length and shows the positional energetic profile derived from alanine scanning.
**FIGURE S4.** Hotspot spacing and clustering by peptide secondary structure. (a) fraction of hotspot pairs separated by short distances (1–2 residues) versus helix‐like periodic spacing (3–4 residues); (b) fraction of adjacent hotspot residues (separation ≤2) grouped by peptide secondary structure.
**FIGURE S5.** The variation of the secondary structure class composition with the peptide length. Bar plots showing the composition of secondary elements in each peptide length range.
**FIGURE S6.** Relationship between intramolecular disulfide bond count and binding energy. Average binding free energy (Δ*G*) of peptide–protein complexes as a function of the number of intramolecular disulfide bridges within the peptide. Points represent mean ΔG values per group, and shaded regions indicate variability within each category.
**FIGURE S7.** Comparing the energy profiles in peptides with (a) and without helical structures (b) at length 17.

## Data Availability

The curated dataset, cluster representatives, supplementary tables, and analysis outputs are deposited in Zenodo under the following DOI: 10.5281/zenodo.19223613. We also developed a comprehensive web interface, available at https://pepdb_curated.crg.eu/, which enables interactive browsing of the curated complexes, structure visualization, display of predicted hotspot residues and interaction energies, and provides ready‐to‐use parameters for running FoldX commands, including RepairPDB, SequenceDetail, AnalyseComplex, and BuildModel.
