# Supplementary material for: A curated structural dataset of peptide–protein complexes reveals biases in existing datasets and principles of peptide binding
Source: Protein Sci. 2026 Sep 15;35(10):e70779. doi: 10.1002/pro.70779 (PMC13575975; doi:10.1002/pro.70779)
Supplement: Supplementary file 1 [file PRO-35-e70779-s001.docx]

Supplementary information

The curated peptide–protein interaction dataset generated in this manuscript is available in Zenodo under the following DOI: [10.5281/zenodo.19223613](https://zenodo.org/records/19223613?preview=1&token=eyJhbGciOiJIUzUxMiIsImlhdCI6MTc3NDg2NzY1NSwiZXhwIjoxNzg1MzY5NTk5fQ.eyJpZCI6ImUwNDVjMjZmLTcwMTItNDNkNC04NTNkLTdlYWVkZDAyMzYzZiIsImRhdGEiOnt9LCJyYW5kb20iOiJmNGYwY2U3ZTllZDQwYWQzNDgzN2JjOWRkNWZkMmVmMiJ9.oz_bfT7i4VbFy74IoHh8h4zKGZY_ey0gKQWPV3ZLFq-gAwNz56_q0Kf5OEFqbuy62BL0a156XQYb5O6OoHQKlg) and a web interface is available at <https://pepdb_curated.crg.eu/> which enables interactive browsing of the curated complexes, structure visualization, display of predicted hotspot residues and interaction energies, and provides ready-to-use parameters for running FoldX commands, including RepairPDB, SequenceDetail, AnalyseComplex, and BuildModel.

This supplementary document provides the full dataset classification, residue-level alanine-scan energetic outputs, and additional analyses supporting the main claims in the manuscript. The supplementary figures are organized from the global distribution of computed energetic effects, to positional distributions of energy profiles for different lengths, and finally to secondary-structure-dependent hotspot organization and length-dependent structural composition of bound peptides.

# Supplementary Notes

## Supplementary Note 1: The energy profiling

Energetic contributions of residues forming the peptides were quantified using the FoldX complex alanine scanning command. Each row in the mutation dataframe in supplementary table 1 corresponds to a single alanine substitution at a specific peptide position and includes the resulting change in binding free energy (ΔΔG) and a binary hotspot annotation.

Each mutation is associated with a peptide chain identifier, the peptide Length, the mutated residue position, the resulting energy difference upon the mutation to alanine (ΔΔG in kcal/mol), and the hotspot binary label (if ΔΔG ≥ 1.5 kcal/mol).

To analyze positional trends, mutations were grouped according to peptide length and sequence position. For each (length, position) pair, we computed:

- Mean and median ΔΔG.
- Standard deviation and standard error of the mean.
- Fractions of mutations classified as hotspots.
- Number of observations (cases) contributing to that position, as shown by the thickness of the profiling plot.

Filtered out poorly supported positions using a minimum observation (set to 10 in our case) threshold to avoid over-interpreting sparse data.

## Supplementary Note 2: Analysis of hotspot spacing patterns

To investigate whether hotspot residues exhibit structural periodicity, hotspot separations were analyzed across peptide secondary-structure classes.

In particular, separations of three to four residues were examined because these distances correspond to the periodicity of residues located on the same face of a helix. The fraction of hotspot pairs exhibiting these separations was calculated for each peptide structural class.

Hotspot clustering was also quantified by computing the fraction of adjacent hotspots separated by two or fewer residues.

# Supplementary Tables

**Supplementary Table 1:** Table containing the classification of all entries of the PepBDB.

**Supplementary Table 2:** Table illustrating the change in binding free energy (ΔΔG) upon mutations to alanine along the chain of the peptide.

**Supplementary Table 3:** Frequency of Hotspots per amino acid type. This table reports the number of hotspot occurrences for each wild-type residue observed in the dataset, together with the total number of mutations and the resulting hotspot fraction.

Supplementary Tables 1- 3 are available in the following document:

[Supplementary_Peptide_Dataset](https://docs.google.com/spreadsheets/d/1Jp52tDeKTIE0e4VnXmMS8-DrP_Xx_fx9EokDEp7QG3M/edit?usp=sharing)

**Supplementary Table 4: First hotspot position with respect to N-termini and C-termini**

Statistics describing the distance of the first hotspot from the N-terminus and C-terminus of peptides. The results indicate that hotspot residues tend to occur within the first few residues from either terminus.

| **Count** | **N-terminus** | **C-terminus** |
| --- | --- | --- |
| **Total** | 2522 | |
| **Mean** | 3.69 | 3.16 |
| **Standard deviation** | 2.41 | 2.13 |
| **Median** | 3 | 3 |

# Supplementary Figures

## Supplementary Figure 1:

To place the hotspot definition threshold in the context of the full dataset, Supplementary Figure 1 shows the global distribution of ΔΔG values from alanine scanning across the curated peptide set, and only for residues that contribute to the interaction (those contacting the protein). The distribution shows that most of these substitutions have modest effects on binding, whereas a smaller subset produces strongly destabilizing changes, consistent with energetically dominant hotspot positions.

**Supplementary Figure. 1:** Histogram of ΔΔG values obtained from alanine scanning across the curated peptide dataset. The vertical dashed line indicates ΔΔG = 1.5 kcal/mol.

## Supplementary Figure 2:

Since positional energetic profiles can be influenced by uneven sampling, Supplementary Figure 2 reports the number of alanine mutations available at each sequence position for representative peptide lengths. This figure serves as a coverage control for the position-dependent analyses presented in the main text.

**Supplementary Figure. 2:** Positional sampling across peptides. Number of alanine mutations available per position for representative peptide lengths (6, 8, and 17 residues). This illustrates the positional coverage of the dataset used for the energy profiling analysis. The numbers change across positions because we don't consider glycine-to-alanine and alanine-to-alanine mutations.

## Supplementary Figure 3:

Supplementary Figure 3 illustrates the positional ΔΔG profiles across all peptide lengths with sufficient support. These profiles highlight the transition from a compact, energetic organization in short peptides to increasingly heterogeneous, multi-peak patterns in longer peptides.

**Supplementary Figure. 3:** Positional ΔΔG profiles by peptide length. Mean ΔΔG values across sequence positions for peptides grouped by length. Each panel corresponds to a specific peptide length and shows the positional energetic profile derived from alanine scanning.

## Supplementary Figure 4:

To test whether hotspot spacing is related to peptide secondary-structure composition, Supplementary Figure 4 compares hotspot clustering and helix-like periodicity across peptide secondary-structure classes. The analysis supports the conclusion that coil and β-sheet peptides favor short-range clustering, whereas helical peptides are enriched for 3–4 residue separations consistent with α-helical presentation.

**Supplementary Figure. 4: Hotspot spacing and clustering by peptide secondary structure.** (A) fraction of hotspot pairs separated by short distances (1–2 residues) versus helix-like periodic spacing (3–4 residues); (B) fraction of adjacent hotspot residues (separation ≤2) grouped by peptide secondary structure.

## Supplementary Figure 5:

Supplementary Figure 5 provides the structural context for the length-dependent analyses by showing how the secondary-structure composition of bound peptides changes across peptide length bins. Very short peptides are predominantly coil-like, whereas helix and β-sheet content increase with length.

**Supplementary Figure. 5: The variation of the secondary structure class composition with the peptide length.** Bar plots showing the composition of secondary elements in each peptide length range

## Supplementary Figure 6:

Supplementary Figure 6 reflects the relationship between the number of intramolecular bridges established within each peptide and the predicted ΔG of interaction per complex involving each peptide. We observed that the increase in the number of intramolecular disulfide bridges is associated with an enhanced stabilization of the peptide fold.

**Supplementary Figure. 6: Relationship between intramolecular disulfide bond count and binding energy.** Average binding free energy (ΔG) of peptide–protein complexes as a function of the number of intramolecular disulfide bridges within the peptide. Points represent mean ΔG values per group, and shaded regions indicate variability within each category.

## Supplementary Figure 7:

Supplementary Figure 7 shows energy profiles for length 17 peptides with and without helical structures. The ~3-4 residue periodicity in hotspot positioning is evident in both cases:

**Supplementary Figure. 7: Comparing the energy profiles in peptides with (Panel A) and without helical structures (Panel B) at length 17.**

This observation definitively establishes that the periodicity is not driven by α-helical structure, but reflects a more fundamental physical principle: Random-Walk Model.

**The random-walk analysis:**

At an unbound, disordered peptide can be approximated as a freely jointed polymer chain. For a 17-residue peptide, the characteristic end-to-end distance follows random-walk statistics [(Alston et al. 2023)](https://paperpile.com/c/f8PeGI/i0kd):

R_ee ~ √N ≈ √17 ≈ 4.1 residues: where R_ee is the end-to-end distance, N is the number of residues, and ν ≈ 0.5 for ideal/unperturbed chains. This characteristic length scale (~4 residues) predicts that when the peptide binds to the protein surface, it must make contact points separated by approximately this distance to maintain configurational accessibility.

Upon binding, the peptide transitions from a high-entropy unbound state to a restricted bound conformation. Rather than concentrating all stabilizing interactions at a single anchor point (hotspot), which would impose an extreme entropic loss, the peptide instead distributes its favorable residues periodically along the sequence. This periodic spacing of hotspots (~every 4 residues) matches the characteristic length scale of the polymer random-walk, allowing the peptide to contact the protein surface at multiple points that are physically separated by the polymer's intrinsic end-to-end distance statistics.
